# Soil Carbon Sequestration: Role of Fe Oxides and Polyphenol Oxidase Across Temperature and Cultivation Systems

**DOI:** 10.3390/plants14060927

**Published:** 2025-03-15

**Authors:** Yuhao He, Zhiyu Wang, Jiayi Zhu, Xiang Lin, Jianying Qi

**Affiliations:** College of Agriculture, South China Agricultural University, Guangzhou 510642, China; 202213150104@stu.scau.edu.cn (Y.H.); 202313150117@stu.scau.edu.cn (Z.W.); aklj2345@126.com (J.Z.); 202313150112@stu.scau.edu.cn (X.L.)

**Keywords:** polyphenol oxidase, Fe oxides, soil organic carbon, organic farming, temperature

## Abstract

The “enzyme latch” and “Fe gate” mechanisms are crucial factors influencing soil carbon sequestration capacity, playing a key role in understanding the dynamic changes in soil organic carbon (SOC). However, there is a lack of research regarding polyphenol oxidase (PPO) activity and the concentration of iron oxides in paddy soils under varying incubating temperatures and cultivation practices. This study was conducted over three years in a double-cropping rice area in southern China, incorporating systematic soil sampling to measure PPO activity, Fe oxide concentration, and basic physicochemical properties. The results showed that temperature did not significantly affect either PPO activity or the concentration of Fe oxides. Additionally, compared to conventional management (CK), organic management led to a decrease in Fe oxides (Fe bound to organic matter, reactive Fe, and total free Fe) by 19.1%, 16.2%, and 13.7%, respectively (*p* < 0.05). At the same time, PPO activity did not show any significant changes. Our results indicated that short-term (5 weeks) incubation temperature did not affect PPO activity or Fe oxides, while organic farming decreased Fe oxides without influencing PPO activity. PPO activity increased with the length of the incubation period.

## 1. Introduction

A significant challenge facing the world today is climate change, which the rapid increase in global greenhouse gas emissions has exacerbated. Soil organic carbon (SOC) is a key component of soil fertility that is crucial for enhancing soil quality and promoting greater agricultural yield [1,2]. Although rice paddies account for only 9% of global farmland, they hold more than 14% of SOC stock [3]. Rice cultivation contributes significantly to greenhouse gas emissions. Enhancing SOC sequestration in paddy fields can both improve soil fertility and mitigate climate change by increasing soil carbon pools [4].

The ‘enzyme latch’ theory suggests that in wetland ecosystems like rice paddies, SOC accumulation occurs when polyphenol oxidase (PPO) activity is restricted, limiting the breakdown of organic matter (OM) and enhancing carbon storage [5,6,7]. In contrast, the “Fe gate” theory proposes that reduced Fe(II) levels may lower phenol oxidase and hydrolase activities, thus counteracting the “enzyme latch” and enhancing SOC preservation [8]. Fe(II) stimulated phenol oxidase activity at the microaerophilic conditions tested. This stimulation is attributed to the catalysis of additional OH radical production, promoting the oxidation of phenolics [9]. Fe oxides contribute to SOC stabilization by forming strong, nondesorbable bonds with organic matter and facilitating co-precipitation mechanisms [10,11,12]. The association of this Fe–ligand complex with OM can greatly increase soil carbon content [13]. It has also been shown that Fe-Al (H)oxides significantly inhibit soil carbon mineralization, and in particular, soils with a high Feo mineral content show a greater potential for carbon sequestration [14]. It is known that amorphous Fe oxide exhibits about six times the carbon sequestration potential of the crystalline state [15]. Fe oxide minerals act as stable sinks for SOC sequestration in peatlands, playing a crucial role in long-term carbon preservation [16]. This interaction can lead to the formation of stable organo-Fe complexes, which are crucial for long-term carbon sequestration in soils [6,7]. As a result, Fe(II) oxidation in soils can fulfill its carbon sequestration potential by interacting with PPOs and forming complexes with SOCs.

Cultivation practices, such as organic and conventional farming, affect soil properties by altering PPO activity and Fe oxide dynamics, ultimately influencing SOC sequestration in paddy fields [2,17]. Organic farming often enhances soil organic matter content and microbial activity, leading to increased PPO activity. In contrast, conventional farming practices, which typically rely on chemical fertilizers, may lead to soil acidification and a decline in soil health, negatively impacting microbial communities and enzyme activities [18]. The application of chemical fertilizers can alter the balance of Fe oxides in the soil, which is essential for binding organic carbon and preventing its loss through leaching or erosion [19]. Fe oxides promote SOC stability by facilitating aggregate formation, which reduces carbon loss and enhances sequestration potential [20]. Enhanced PPO activity in organic systems can lead to increased oxidation of phenolic compounds, which are important for the formation of stable OM [21]. This process is further supported by the presence of Fe oxides, which can adsorb organic compounds and protect them from microbial degradation [22]. Research indicates that the long-term application of organic amendments, such as compost or manure, can improve soil structure and increase the availability of nutrients, which in turn supports higher microbial biomass and activity [23]. This positive feedback loop enhances the soil’s capacity to sequester carbon, making organic farming a more sustainable option for maintaining soil health and mitigating climate change impacts in paddy fields [24].

Temperature plays a critical role in influencing soil biochemical processes. Temperature changes can alter the activity of PPOs, potentially accelerating the decomposition of OM and reducing soil carbon sequestration capacity. This is particularly relevant in the context of climate change, where rising temperatures could enhance enzyme activity and lead to increased carbon release from soils [25]. This increase in PPO activity is often linked to changes in microbial community structure and function. The enhanced activity of PPOs under warming conditions can be attributed to increased microbial activity and enzyme production, which are stimulated by higher temperature [26,27]. Higher temperatures can promote the reduction in Fe oxides, leading to the release of bound organic carbon and thus reducing the soil’s carbon sequestration potential. This dynamic is especially pronounced in environments that experience periodic wetting and drying cycles, which can lead to redox fluctuations that destabilize Fe-bound carbon [28,29]. Moreover, elevated temperatures can increase soil respiration rates, leading to greater carbon dioxide emissions. In humid environments, where soils are often waterlogged, temperature-induced changes in redox conditions can significantly impact the cycling of Fe and carbon, potentially leading to increased carbon losses [30,31]. In particular, higher temperatures accelerate microbial respiration and oxygen depletion, causing flooded soils to enter a hypoxic state more quickly [32]. This causes the reduction of Fe(III) to Fe(II), and the accumulation of Fe(II) nonenzymatically breaks down recalcitrant organic carbon (e.g., lignin fragments), leading to the release of CO_2_ [31]. Additionally, the role of temperature in influencing microbial community composition and activity cannot be overlooked. For instance, warming can lead to a decrease in microbial biomass carbon and reduced microbial carbon use efficiency, which can further contribute to the loss of soil carbon under elevated temperatures [33,34].

In summary, the choice between organic and conventional farming and temperature effects has profound implications for soil enzyme activities, Fe oxide dynamics, and soil carbon sequestration in paddy ecosystems. Understanding these interactions is essential for developing sustainable agricultural practices that enhance soil fertility and carbon storage.

Although existing studies have separately explored the effects of “enzyme latch” and “iron gate” mechanisms on SOC sequestration, their interactions under different temperatures and tillage practices have not been systematically analyzed. For example, previous studies have mostly focused on the effects of single factors (e.g., temperature or cultivation practices) on SOC and Fe oxides or on long-term (>10 years) management effects only, ignoring the combined effects of short-term (e.g., 5 weeks) temperature fluctuations and organic/conventional farming on Fe oxide dynamics and PPO activity. Furthermore, the compensatory effect of short-term organic management on Fe-oxide-mediated carbon stabilization potential in a double-cropped rice system remains unclear. In this study, we quantified the synergistic or antagonistic effects of PPO activity and Fe oxides on SOC sequestration in a subtropical double-cropped rice system by controlling the temperature gradient (15 °C–30 °C) and tillage practices, which fills the research gap of multifactorial interaction mechanisms in short-term dynamics. The present study was conducted on a double-cropped rice system under organic farming conditions and aimed to investigate the effects of soil Fe oxides and PPO activity on soil carbon sequestration at different temperatures and cultivation methods. The objectives of the study are the following: (1) quantify how temperature and cultivation practices synergistically regulate Fe oxide dynamics, which stabilize SOC through adsorption and co-precipitation; (2) evaluate temperature-dependent shifts in PPO activity and their implications for SOC decomposition under organic and conventional farming; and (3) assess the net effect of these interactions on SOC sequestration, testing whether organic inputs compensate or synergize SOC sequestration by enhancing carbon inputs.

## 2. Results

### 2.1. Effect of Temperature and Cultivation Methods on PPO Activity

**Figure 1:**
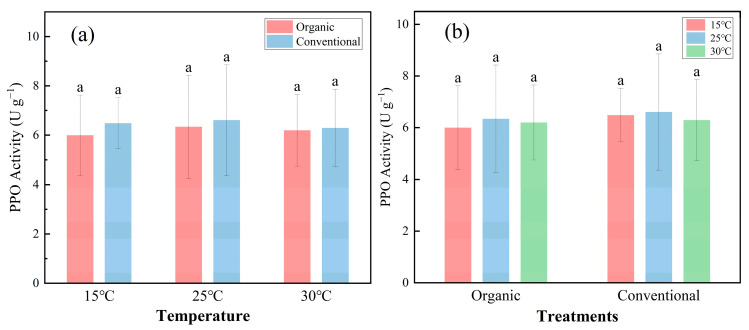
Effect of temperature and cultivation method on PPO activity. (**a**) represents the effect of organic and conventional cultivation on PPO activity at different temperatures. (**b**) represents the effect of individual temperatures on PPO activity under two cultivation methods. Organic: organic farming management; Conventional: conventional farming management. Within a given fraction, bars with the same lowercase letter at the top do not differ at *p* = 0.05. Error bars in the figure represent the standard deviation. (*n* = 90).

In this study, three experimental temperatures—15 °C, 25 °C, and 30 °C—were set and analyzed. Figure 1 illustrates the dynamics of PPO activity under varying temperatures (15 °C, 25 °C, and 30 °C) and cultivation methods (organic vs. conventional). In the organic systems, the PPO activity followed a weak temperature-dependent pattern, peaking at 25 °C (6.34 ± 2.08 U g^−1^) compared to 15 °C (6.00 ± 1.63 U g^−1^) and 30 °C (6.20 ± 1.45 U g^−1^) (Figure 1a). Conversely, in conventional farming, the PPO activity decreased slightly with increasing temperatures (15 °C: 6.48 ± 1.04 U g^−1^; 30 °C: 6.29 ± 1.57 U g^−1^) (Figure 1b). However, statistical analysis (Tukey’s test, *p* > 0.05) indicated no significant differences in the PPO activity across temperature treatments within either cultivation method. Across all temperatures, the PPO activity was consistently lower under organic farming (6.18 ± 1.71 U g^−1^) than under conventional farming (6.46 ± 1.66 U g^−1^) (Figure 1b). However, statistical analysis (*p* > 0.05 for 25 °C and 30 °C) confirmed that these differences were not significant.

**Figure 2:**
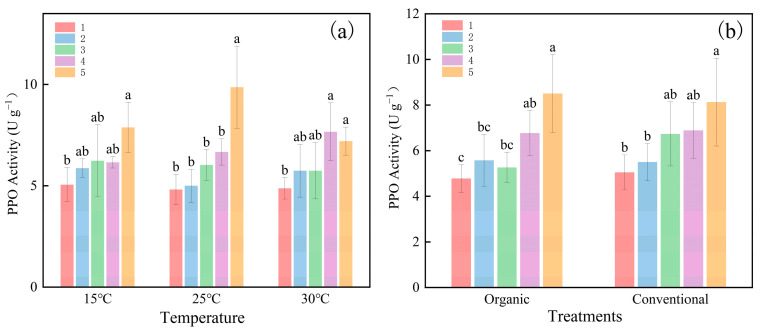
Effect of different culturing times on the activity of PPO. (**a**) represents the activity of PPO at different temperatures for each culturing time. (**b**) represents the activity of PPO at each culturing time under different cultivation methods. Organic: organic farming management; Conventional: conventional farming management. The different numbers represent the order in which the samples were taken, five times in total. Bars with the same lowercase letter at the top do not differ at *p* = 0.05. Error bars in the figure represent the standard deviation. (*n* = 90).

**Figure 3:**
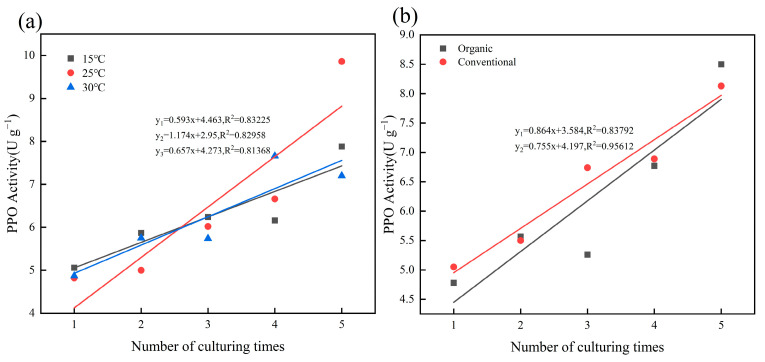
Correlation between different culturing times and PPO activity. (**a**) represents the relationship between different temperatures; (**b**) represents the relationship between different cultivation methods.

Figure 2 illustrates the temporal evolution of the PPO activity over five weeks. The PPO activity increased under all conditions, with a significant rise (*p* < 0.05) from the initial to the final measurement. At 25 °C, the PPO activity increased by 104.9% compared to 55.9% at 15 °C and 47.8% at 30 °C (Figure 2a). In contrast, the PPO activity under organic farming increased by 77.8% compared to 60.9% in the CK (Figure 2b). Figure 3 further confirms a significant positive correlation (*p* < 0.05) between the culturing time and PPO activity across different temperatures and cultivation methods. Notably, at 25 °C, the PPO activity exhibited a steeper temporal slope (1.174) compared to 15 °C (0.593) and 30 °C (0.657) (Figure 3a). Similarly, there was a greater trend of increasing PPO activity over time under organic farming (slope = 0.864) compared to the CK (slope = 0.755). These findings indicate that prolonged incubation enhances PPO activity, particularly under moderate temperatures and organic regimes, potentially accelerating organic matter decomposition and reducing SOC sequestration efficiency.

### 2.2. Effect of Temperature and Cultivation Methods on Fe Oxide Concentration

**Figure 4:**
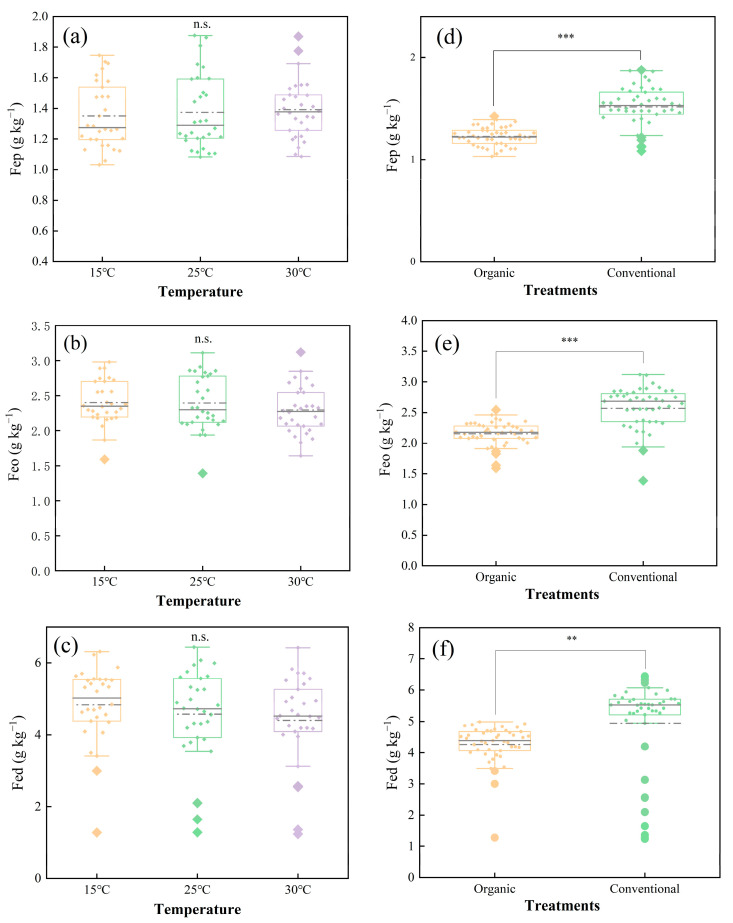
Effect of temperature and cultivation method on iron oxide concentration. (**a**–**c**) represent the effect of different temperatures on the contents of Fep, Feo, and Fed. (**d**–**f**) represent the effect of different cultivation methods on the contents of Fep, Feo, and Fed. Organic: organic farming management; Conventional: conventional farming management. The value bars with ns are not significant; asterisk denotes statistically significant difference. n.s. *p* > 0.05; ** *p* < 0.01; *** *p* < 0.001. Error bars in the figure represent the standard deviation. (*n* = 90 per Fe oxide fraction).

Figure 4 delineates the response of the Fe oxides (Fep, Feo, and Fed) to temperature and cultivation. The temperature had no statistically significant effect on Fe oxide concentrations (*p* > 0.05) (Figure 4a–c). The cultivation methods, however, profoundly altered the Fe oxide dynamics. Organic farming reduced the Fep, Feo, and Fed concentrations by 19.1%, 16.2%, and 13.7%, respectively (*p* < 0.05) (Figure 4d–f).

**Figure 5:**
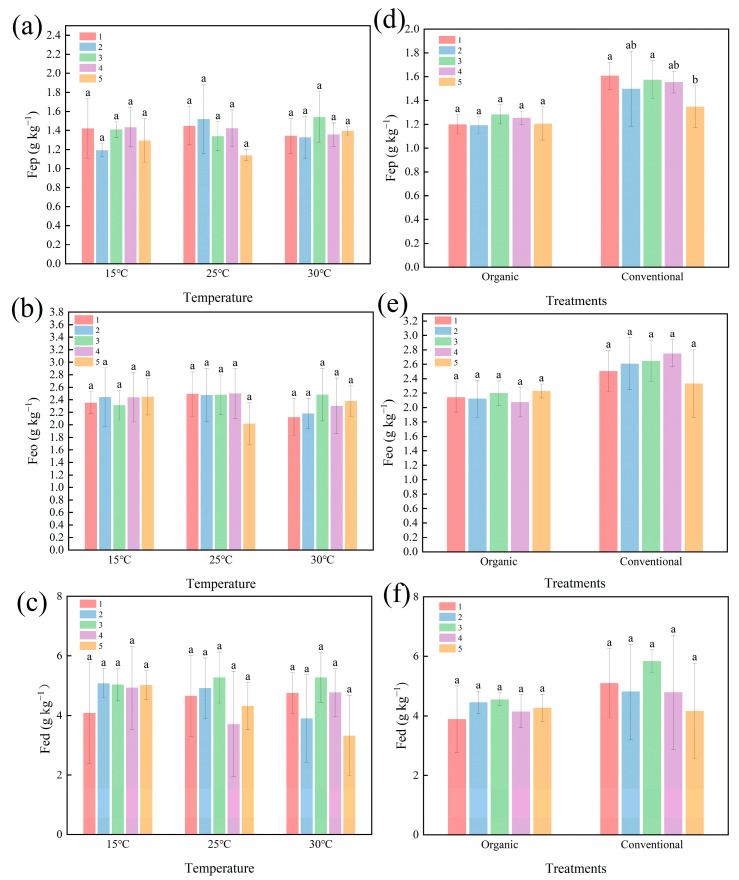
Effect of different culturing times on the activity of iron oxides. (**a**–**c**) represent the effect of culturing time at different temperatures on Fep, Feo, and Fed. (**d**–**f**) represent the effect of culturing time on Fep, Feo, and Fed under different cultivation methods. Organic: organic farming management; Conventional: conventional farming management. The different numbers represent the order in which the samples were taken, five times in total. Bars with the same lowercase letter at the top do not differ at *p* = 0.05. Error bars in the figure represent the standard deviation. (*n* = 90 per Fe oxide fraction).

Figure 5 further reveals minor fluctuations in the Fe oxides over five weeks. The contents of Fep, Feo, and Fed fluctuated with longer incubation times at different temperatures, but this change was not significant (Figure 5a–c). Likewise, the Fe oxide concentrations fluctuated across cultivation methods, but these changes were also not statistically significant (*p* > 0.05) (Figure 5d–f). Under organic farming, the Fep concentration remained stable, with a minor, nonsignificant change from 1.2% in week 1 to 1.21% in week 5. A notable exception was observed under conventional farming, where the Fep concentration significantly decreased from 1.61% to 1.35% (16.1% reduction, *p* < 0.05). This may be attributed to transient redox shifts or microbial Fe mobilization. This significant change highlights the importance of higher temporal resolution in future studies to capture Fe mobilization dynamics.

### 2.3. Integrated Analysis of Soil Physicochemical Properties

Further analysis of the soil physicochemical properties (Table S1) revealed that organic farming significantly increased the SOC content (16.70 ± 1.50 g kg^−1^) compared to conventional farming (13.48 ± 0.67 g kg^−1^) despite lower Fe oxide concentrations. Notably, the total nitrogen (1.68 ± 0.18 vs. 1.37 ± 0.05 g kg^−1^) and available nitrogen (122.02 ± 6.16 vs. 97.25 ± 7.85 mg kg^−1^) were also higher in organic systems, likely promoting microbial activity and carbon turnover. Conversely, conventional farming exhibited higher total phosphorus (0.56 ± 0.002 vs. 0.48 ± 0.022 g kg^−1^) and available potassium (60.67 ± 0.88 vs. 39.00 ± 1.53 mg kg^−1^). The soil pH under both cultivation methods was also relatively close, with little variation (organic: 5.23 ± 0.03; CK: 5.08 ± 0.07). Additionally, the clay content remained consistent between the organic (20.4% ± 0.7%) and conventional (20.0% ± 0.3%) systems. 

## 3. Discussion

### 3.1. The Role of PPO in SOC Stabilization

In this study, it was found that the PPO activity demonstrated a trend influenced by varying temperatures and cultivation practices, although this change was not significant. This reflects the inability to reduce SOC mineralization by inhibiting PPO activity under the currently set temperature range and cultivation methods. The three temperatures set in this experiment are in a more general range of climatic environments and did not show significant changes from 15 °C to 30 °C. It is possible that the temperature still has not reached the critical value that can significantly affect its activity [35,36].

Our data also show that the activity of PPOs becomes significantly elevated with longer culturing time, and this effect was more pronounced at 25 °C and in the case of organic farming. Thus, it appears that temperature and cultivation practices affect soil PPO activity in short-term culturing indirectly by influencing soil dynamics. Previous studies have also shown that the incubation temperatures can influence the metabolic activity and diversity of soil microorganisms, with different temperatures favoring different microbial communities [37]. This can lead to variations in enzyme activities, including PPOs, as different microbial taxa may have distinct enzymatic capabilities. Furthermore, studies have demonstrated that organic amendments can enhance microbial biomass and enzyme activities, including PPOs, by providing additional nutrients and organic matter for microbial growth [38]. This suggests that temperature management, alongside organic farming, can be an indirect strategic approach to regulate PPO activity.

These data help to characterize the changes in soil PPO activity in the short term and at current more-normal temperatures. However, there are some limitations to this procedure: (1) This experiment specifically measured short-term PPO activity patterns. (2) The selected temperature range (three levels) may not have encompassed the threshold required to significantly influence PPO activity, particularly given that existing thermal studies on soil enzymes typically employ higher temperature gradients. (3) Repeated sampling from the same incubation system across five timepoints might have induced cumulative environmental disturbances in the soil matrix, potentially obscuring significant variations in the PPO activity measurements.

### 3.2. The Role of Fe Oxides in SOC Stabilization

Our results highlight that organic farming enhances SOC stocks despite reduced Fe oxide content, suggesting a compensatory role of organic amendments in carbon stabilization. Compared to conventional farming, organic farming significantly reduced the Fe oxide content (Figure 4d–f), which may be related to the promotion of the Fe reduction process by organic matter input [39]. Even though Fe oxides can fix SOC by adsorption or co-precipitation [10], the SOC levels in organic systems were higher than in conventional systems (Table S1), suggesting that exogenous organic matter (e.g., compost) directly increases carbon inputs and compensates for the reduced sequestration potential of Fe oxides. This finding contradicts the results of long-term fertilization trials [15]. It may stem from the short-term nature of this study (3 years) that did not sufficiently stimulate the Fe oxide–organic matter stabilization process. Thus, short-term organic management relies more on organic matter accumulation than Fe-mediated carbon sequestration, while long-term effects need to be further verified.

Our results challenge the conventional view that Fe oxides are indispensable for carbon stabilization. This paradox may stem from the dual function of organic amendments: (1) They enhance microbial activity, promoting Fe reduction and decreasing Fe oxide-bound carbon [39,40,41], and (2) fresh organic inputs (e.g., compost) provide labile carbon, which is either rapidly assimilated by microbial biomass or physically stabilized within aggregates [42]. This highlights the importance of considering the interactions between organic amendments, microbial communities, and Fe oxides in agricultural systems [43,44]. Notably, our short-term experiment (3 years) may prioritize the latter process, whereas long-term organic management (e.g., >30 years) could reveal cumulative Fe oxide depletion risk. Therefore, balancing organic inputs with Fe oxide preservation is critical for sustainable carbon sequestration.

It was also found that there was no significant effect of temperature on the Fe oxides (Fed, Feo, and Fep) in the short term. This finding aligns with previous studies that have explored the stability and transformation of Fe oxides under varying environmental conditions. For instance, the stability of Fe oxides in soil aggregates is often driven by the interaction with other soil constituents rather than temperature fluctuation [45]. This is also evidenced by the above discussion on organic farming. Additionally, the presence of organic matter and mineral surfaces can significantly influence the oxidation rates and stability of Fe oxides, further supporting the notion that temperature alone may not be a dominant factor in short-term transformations [46]. Taken into consideration, long-term experiments should be carried out to further investigate the dynamic effects of temperature and cultivation methods on Fe oxides.

### 3.3. Integrated Consideration of PPO Activity and Fe Oxides in Carbon Sequestration

Our results revealed a paradoxical relationship between Fe oxides and SOC under organic farming: despite significantly lower concentrations of Fep, Feo, and Fed, the SOC content was higher in organic systems compared to conventional management. This suggests that organic amendments (e.g., compost) compensate for reduced Fe-mediated carbon stabilization by increasing labile carbon pools and enhancing microbial biomass. These effects may temporarily outweigh Fe oxide-driven carbon adsorption.

Critically, the lack of temperature effects on the Fe oxides aligns with studies emphasizing that Fe stability in soils is more influenced by organic–mineral interactions than short-term thermal fluctuations. However, the observed increase in PPO activity with prolonged incubation highlights a potential risk: elevated PPO activity under organic farming at 25 °C could accelerate phenolic compound degradation, theoretically destabilizing the SOC. This risk arises because of the phenolic compounds, which are critical for stabilizing the SOC through their inhibitory effects on hydrolytic enzymes. According to the “enzyme latch” theory, this would release the inhibition of hydrolytic enzymes by phenolic compounds, which would allow for accelerated mineralization of the SOC. Yet, our data showed no decline in SOC under organic management, implying that the benefits of organic inputs (e.g., enhanced microbial carbon use efficiency and aggregate protection) counterbalance PPO-driven mineralization [21,22].

This duality underscores the need to distinguish between short-term and long-term dynamics. While organic farming currently relies on exogenous organic matter to boost the SOC, prolonged Fe oxide depletion may gradually weaken the “Fe gate” mechanism, necessitating balanced management practices. Future studies should prioritize long-term field trials to evaluate whether the SOC gains from organic inputs can persist without Fe oxide support, particularly under climate-driven temperature shifts.

## 4. Materials and Methods

### 4.1. Experimental Site

This study is based on a three-year field trial conducted from 2021 to 2023 in Huangjiashan Village, Luoping Town, Yunfu City, Guangdong Province, China (22.62° N, 111.57° E). The three-year period was selected to capture initial changes in SOC, Fe oxides, and enzyme activity under organic/conventional farming, as shorter-term studies often fail to detect meaningful shifts in these parameters.

The experimental site was located in a subtropical monsoon climate zone with an average annual maximum temperature of 27 °C, minimum temperature of 18 °C, and annual precipitation of 1026.4 mm. The field was planted with *Xiangyaxiangzhan*, a fragrant rice variety widely grown in southern China, using a two-season rice cropping system with an early and late rice season. The early rice season was sown in March, transplanted in April, and harvested in July. The late rice season was sown in July, transplanted in August, and harvested in November. Before the experiment started, the organic matter content was 30.1 g kg^−1^, total nitrogen was 0.891 g kg^−1^, total phosphorus was 0.45 g kg^−1^, and total potassium was 19.2 g kg^−1^. We also detected the soil’s basic properties in 2023, as shown in Table S1.

### 4.2. Experimental Design

The project included two types of rice management: conventional farming (CK) and organic farming. The conventional farming model used a chemically intensive approach using a commercial inorganic fertilizer formulation consisting of 15% urea nitrogen, 5% P_2_O_5_, and 15% K_2_O applied at a rate of 900 kg ha^−1^, with 60% used as a basal fertilizer and 40% as a follow-up fertilizer at the tillering stage. In the conventional mode, 30 g ha^−1^ acetamiprid and 20 g ha^−1^ avermectin were used for total pest control. For weed control, 500 mL ha^−1^ of fluroxypyr-methyl and 20 g ha^−1^ of deltamethrin were used, while for rice disease control, 30 kg ha^−1^ of hymexazol and 100 g ha^−1^ of kasuga-mycin were used.

In contrast, the management strategy for organic farming, based on the principles of sustainability and environmental protection, prioritized the use of organic slow-release fertilizer. It was composed of 10% total nitrogen, 3% total potassium, and up to 55% organic matter. This mixture was applied at a rate of 1500 kg ha^−1^. Weed control in the organic system was managed through manual weeding. Pest and disease management was managed through the use of biological agents such as *Bacillus subtilis* (4.5 L ha^−1^ at a concentration of 8000 IU μL^−1^), matrine, *Bacillus thuringiensis*, and Mamestra brassicae Nuclear Polyhedrosis Virus.

The experiment was conducted in a randomized complete block design with three replications, 40 m^2^ (5.0 m × 8.0 m) per plot, and two soil samples were collected from each plot for a total of 12 soil samples.

Field-collected soil samples from organic and conventional plots were homogenized separately to form two composite samples. From each composite, 100 g of air-dried soil was transferred into sterile glass jars (n = 6 total: 2 cultivation methods × 3 temperatures). Each jar received 30 mL of deionized water to simulate paddy field moisture conditions and was pre-incubated for 5 days at respective temperatures (15 °C, 25 °C, 30 °C), which aims to stabilize microbial activity, reducing transient effects caused by abrupt environmental changes. This approach is particularly effective in ensuring that the microbial communities in the soil reach a stable state before any experimental treatments are applied. Adaptation of soil microorganisms to temperature changes suggests that short-term incubation helps stabilize microbial communities [32]. It was based on the average temperature of the experimental site and the climatic conditions in Guangdong province (Winter, 10 °C–20 °C; Spring/Autumn, 20 °C–28 °C and Summer, exceeding 30 °C). By selecting 15 °C (representing cooler winter conditions), 25 °C (mild transitional seasons), and 30 °C (summer temperatures), we aimed to simulate realistic temperature scenarios encountered in local paddy systems.

Following a 5-day pre-incubation to stabilize microbial activity, soils were sampled weekly for five weeks (weeks 1–5). The 5-week sampling period was designed to capture short-term dynamics of PPO activity and Fe oxide responses. Prior studies on soil enzyme kinetics have demonstrated that microbial communities and enzyme activities often exhibit shifts within weeks to months under controlled conditions [47]. The first sampling occurred one week after pre-incubation (week 1), with subsequent samplings at weekly intervals (weeks 2–5). This design prioritized short-term responses under stabilized conditions, as baseline sampling was omitted to avoid confounding effects from initial soil disturbance. The incubation system maintained constant humidity and darkness to minimize photodegradation and evaporation effects.

### 4.3. PPO Activity Analysis

PPO activity was determined by the colorimetric method. First, 1% catechol solution was prepared, 1 g of catechol (pyrogallic gallic acid) was dissolved in distilled water, and the volume was fixed to 100 mL; pH = 4.5 citric acid–phosphoric acid buffer was prepared, 35.61 g of disodium phosphate was fixed to 1 L, 21.01 g of citric acid was fixed to 1 L, and 9 mL of disodium phosphate was mixed with 11 mL of citric acid, which was the citric acid–phosphoric acid buffer with pH = 4.5. A pH meter was needed, and if the actual pH value was slightly deviated, the pH value was determined by a colorimetric method. The buffer needs to use a pH meter; if the actual value of pH has a slight deviation, it needs to be supplemented according to the actual pH value of the corresponding solution to adjust the pH to 4.5; the preparation of the standard solution, that is, potassium dichromate solution (5 mmol/L), took 2.9419 g potassium dichromate that was fixed to 2 L to finally obtain the ethyl ether solution.

The visible spectrophotometer was preheated for more than 30 min, the wavelength was adjusted to 430 nm, and the ether reagent was zeroed. The standard solution was diluted to 0.2 mg/mL, 0.1 mg/mL, 0.05 mg/mL, 0.025 mg/mL, 0.0125 mg/mL, 0.00625 mg/mL, 0.003125 mg/mL, and 0 mg/mL with 0.5 mol/L HCl solution. An amount of 1000 μL of the standard was needed for the test. An amount of 0mg/mL standard solution was used to adjust the zero value, and 1mL of the diluted standard solution was taken into 1 mL glass cuvette to measure m at 430 nm. The absorbance value A was measured at 430 nm, and the standard curve was made according to the absorbance (x) and concentration (y, mg/mL).

We added 500 μL of reagent I (1% catechol solution) into each centrifuge tube, covered the centrifuge tubes, shook and mixed well, and reacted the solution in a water bath at 30 °C for 1 h. We removed the samples that had been reacted for 1 h from the water bath, added 200 μL of citrate–phosphate buffer at pH = 4.5 to the centrifuge tubes, added 1750 μL of ether and operated the solution in a ventilated environment, and covered the centrifuge tubes with caps. We centrifuged the tube lid, shook the tube several times, let it stand at room temperature for 30 min, took 0.2 mL of the upper layer of the liquid in a 96-well quartz enzyme plate (3 technical replicates), and measured the absorbance value at 430 nm. If the difference in absorbance value was too large, we continued to measure for 1–2 replicates and recorded the data with the closest value. The finished plate should be rinsed and dried after disposing of the measurement solution to ensure that the absorbance of each well of the blank plate is close to the absorbance before the next measurement. According to the standard curve, the sample absorbance value A (x) was substituted into the formula to calculate the sample concentration y (mg/mL), and the production of 1 mg of purple gall tannin per g of soil sample per day was defined as a unit of enzyme activity. The PPO activity was calculated using the following formula:

$$A-ppo=\frac{y\times V_{phase\;extraction}}{W\times T}$$

where *y* is the concentration of the sample calculated from the standard curve (mg/mL), *V_phase extraction_* is the volume of the extraction phase solution (mL), *W* is the mass of the sample (g), and *T* is reaction time (d).

### 4.4. Analysis of Soil Fe Oxide Content

Application of sodium pyrophosphate method for the analysis of Fe bound to organic matter (Fep) in soil samples is described as follows: Weigh 0.300 g of air-dried soil (<2 mm), grind it through a 0.15 mm (100 mesh) sieve, and place it in a 50 mL centrifuge tube with a screw cap. Add 30 mL of 0.1 mol·L^−1^ sodium pyrophosphate solution to the above centrifuge tube and tighten the lid. The centrifuge tube was placed in a rotary shaker and shaken overnight (16 h). An amount of 0.5 mL of 0.1% flocculant was added and centrifuged at 510 g (force) for 10 min, and it was necessary to stir all the time when the flocculant was added. It was then filtered using a 0.025 μm ultrafiltration microporous filter. The supernatant was taken and analyzed in time, and if the filtrate was turbid, it needed to be filtered again. We determined the Fep content using atomic absorption spectrometry (AAS). The concentration of Fep was calculated by applying the following equation:

$$Fep(\%)=\frac{C\times V}{m\times 1000}$$

where C is the concentration of the solution (μg/mL), V is the volume of the extraction solvent (mL), and m is the mass of the sample (g).

Application of acidic ammonium oxalate method (protected from light) for the analysis of reactive Fe (Feo) in soil samples is described as follows: Weigh 0.250 g of air-dried soil (<2 mm), grind it through a 0.15 mm (100 mesh) sieve, and load it into a 15 mL lightproof centrifuge tube with a screw cap. Add 10 mL of 0.2 mol L^−1^ acidic ammonium oxalate solution to the above centrifuge tube and tighten the lid. Place the centrifuge tube in a rotary oscillator and oscillate under dark conditions for 4 h (180 r min^−1^). Centrifuge at 510 g (force) for 20 min and take the supernatant for analysis. The supernatant needs to be stored away from light to avoid photolysis of the oxalate and precipitation of dissolved metals, as in the determination of Fe by atomic absorption spectrometry (AAS). The concentration of Feo was calculated by applying the following equation:

$$Feo(\%)=\frac{C\times V\times D}{m\times 1000}$$

C is the concentration of the solution (μg/mL), V is the volume of the extraction solvent in milliliters (mL), D is the dilution factor (unitless), and m is the mass of the sample (g).

Application of the sodium dithionite–citric Acid method for the analysis of total free Fe (Fed) in soil samples is described as follows: Weigh 0.500 g of air-dried soil (<2 mm), grind it through a 0.15 mm (100 mesh) sieve, fill a 50 mL plastic centrifuge tube with a screw cap, add 25 mL of 0.68 mol L^−1^ sodium citrate solution, and add 0.4 g of sodium dithionite (using a calibrated spoon). The centrifuge tube was tightened and shaken in a rotary shaker overnight (~16 h). The cap of the centrifuge tube was removed and centrifuged at 510 g (force) for 20 min, and the Fe content of the filtrate was determined by atomic absorption spectrometry (AAS). The concentration of Fed was calculated by applying the following equation:

$$Fed(\%)=\frac{C\times V\times D}{m\times 1000}$$

C is the concentration of the solution (μg/mL), V is the volume of the extraction solvent in milliliters (mL), D is the dilution factor (unitless), and m is the mass of the sample (g).

### 4.5. Statistical Analysis

All statistical analyses were conducted using Origin 2024. A one-way analysis of variance (ANOVA) was performed to assess the effects of different temperatures, cultivation methods, and culture durations on PPO activity and Fe oxide content. Following ANOVA, Tukey’s post hoc test was employed to identify significant differences between treatment means (*p* < 0.05). This post hoc analysis was facilitated by the Paired Comparison plugin within Origin 2024 and through this plugin to generate box line charts and bar charts.

For clarity, each Fe oxide fraction (Fep, Feo, Fed) and PPO activity dataset comprised 90 independent measurements (3 temperatures × 2 cultivation methods × 5 sampling times × 3 replicates). All figures explicitly state the sample size (n) for each analysis.

## 5. Conclusions

After three years of organic and conventional rice farming management in South China, we analyzed soil PPO activity and Fe oxide content under two cultivation methods, examining how these two indicators changed with varying temperatures in controlled conditions. The results showed that the concentration of iron oxides was significantly lower under organic farming compared to CK. This suggests that conventional agriculture, with its higher Fe oxide content, could theoretically fix more soil organic carbon through adsorption or co-precipitation over time. However, organic farming enhanced SOC stocks despite reducing Fe oxide content, primarily through direct organic matter inputs that offset the weakened Fe-mediated carbon stabilization. Short-term temperature variations (15–30 °C) did not significantly alter PPO activity or Fe oxide dynamics, suggesting that Fe stability is governed more by organic–mineral interactions than thermal fluctuations. However, the observed increase in PPO activity over time, particularly at 25 °C under organic management, highlights a potential trade-off: while organic amendments currently sustain SOC via some mechanisms, long-term Fe oxide depletion could gradually undermine the “Fe gate” effect. Moreover, temperature seems to tend to indirectly alter PPO activity during its dynamic incubation, potentially exacerbating SOC loss. Thus, organic farming practices should be complemented with strategies to preserve Fe oxides to ensure sustainable carbon sequestration. Future research must address these interactions through extended field trials, and climate-resilient agricultural designs and long-term experiments are needed to explore these dynamic changes further.

## Data Availability

The raw data supporting the conclusions of this article will be made available by the authors upon request.

## Supplementary Materials

The following supporting information can be downloaded at https://www.mdpi.com/article/10.3390/plants14060927/s1. Table S1: Soil basic properties under organic farming and conventional farming managements. Means and standard errors are shown. CK represents the traditional farming management. Lowercase letters indicate a significance level where *p* < 0.05.

## Abbreviations

The following abbreviations are used in this manuscript:

SOC	Soil organic carbon
PPO	Polyphenol oxidase
CK	Control check
Fep	Fe bound to organic matter.
Feo	Reactive Fe
Fed	Total free Fe
OM	Organic matter
