# Supplementary material for: Soil Carbon Sequestration: Role of Fe Oxides and Polyphenol Oxidase Across Temperature and Cultivation Systems
Source: Plants (Basel). 2025 Mar 15;14(6):927. doi: 10.3390/plants14060927 (PMC11945063; doi:10.3390/plants14060927)
Supplement: Supplementary file 1 [file plants-14-00927-s001.zip › plants-3524373-supplementary.pdf]

# Supporting information of "Soil Carbon Sequestration: Role of Fe Oxides and Polyphenol Oxidase Across Temperature and Cultivation Systems"

**Yuhao He#, Zhiyu Wang#, Jiayi Zhu#, Xiang Lin# and Jianying Qi ***

College of Agriculture, South China Agricultural University, Guangzhou, 510642, China;202213150104@stu.scau.edu.cn(Y.H.)
* Correspondence: qi@scau.edu.cn (J. Q.).
# These authors contributed equally to this work

**Table S1.** Soil basic properties under organic farming and conventional farming managements. Means and standard errors are shown. CK represents the traditional farming management. Lowercase letters indicate a significance level where P < 0.05.

| Soil properties | Soil organic carbon ($g\ kg^{-1}$) | Soil total nitrogen ($g\ kg^{-1}$) | pH | Clay |
|---|---|---|---|---|
| Organic farming | 16.70± 1.50a | 1.68± 0.18a | 5.23± 0.03a | 20.4%± 0.7%a |
| CK | 13.48± 0.67a | 1.37± 0.05a | 5.08± 0.07a | 20.0%± 0.3%a |

| Soil properties | Total phosphorus ($g\ kg^{-1}$) | Total potassium ($g\ kg^{-1}$) | Available nitrogen ($mg\ kg^{-1}$) | Available potassium ($mg\ kg^{-1}$) |
|---|---|---|---|---|
| Organic farming | 0.48± 0.022b | 9.21± 0.152a | 122.02± 6.159a | 39.00± 1.528b |
| CK | 0.56± 0.002a | 8.13± 0.096b | 97.25± 7.849a | 60.67± 0.882a |
